# Translation, cross-cultural adaptation to Brazilian- Portuguese and reliability analysis of the instrument Rapid Entire Body Assessment-REBA

**DOI:** 10.1590/bjpt-rbf.2014.0035

**Published:** 2014

**Authors:** Andressa M. Lamarão, Lucíola C. M. Costa, Maria L. C. Comper, Rosimeire S. Padula

**Affiliations:** 1 Master and Doctorate Program in Physical Therapy, Universidade Cidade de São Paulo (UNICID), São Paulo, SP, Brazil; 2 Physical Therapy Program, União Metropolitana de Ensino e Cultura, Itabuna, BA, Brazil

**Keywords:** biomechanical, occupational health, ergonomics, physical therapy

## Abstract

**Background:**

Observational instruments, such as the Rapid Entire Body Assessment, quickly assess biomechanical risks present in the workplace. However, in order to use these instruments, it is necessary to conduct the translational/cross-cultural adaptation of the instrument and test its measurement properties.

**Objectives:**

To perform the translation and the cross-cultural adaptation to Brazilian-Portuguese and test the reliability of the REBA instrument.

**Method:**

The procedures of translation and cross-cultural adaptation to Brazilian-Portuguese were conducted following proposed guidelines that involved translation, synthesis of translations, back translation, committee review and testing of the pre-final version. In addition, reliability and the intra- and inter-rater percent agreement were obtained with the Linear Weighted Kappa Coefficient that was associated with the 95% Confidence Interval and the cross tabulation 2×2.

**Results:**

: The procedures for translation and adaptation were adequate and the necessary adjustments were conducted on the instrument. The intra- and inter-rater reliability showed values of 0.104 to 0.504, respectively, ranging from very poor to moderate. The percentage agreement values ranged from 5.66% to 69.81%. The percentage agreement was closer to 100% at the item 'upper arm' (69.81%) for the Intra-rater 1 and at the items 'legs' and 'upper arm' for the Intra-rater 2 (62.26%).

**Conclusions:**

The processes of translation and cross-cultural adaptation were conducted on the REBA instrument and the Brazilian version of the instrument was obtained. However, despite the reliability of the tests used to correct the translated and adapted version, the reliability values are unacceptable according to the guidelines standard, indicating that the reliability must be re-evaluated. Therefore, caution in the interpretation of the biomechanical risks measured by this instrument should be taken.

## lntroduction

The Work-related Musculoskeletal Disorders (WMSDs) represent an important health problem in Brazil^1^. Records have shown a 600% increase in the number of cases of MSDs between the years of 1998 and 2005^2^. The causes of WMSDs are complex due to the numerous risk factors involved^3^, among which are: biomechanical risks related to the musculoskeletal overload imposed by labour tasks, the use of excessive force, repetitive motion, improper positioning and prolonged static postures associated with the intensity, speed and duration of the exposure^4^.

To understand the influence of risk factors in the emergence of WMSDs, an evaluation of the work environment using appropriate ergonomic approaches, such as observational instruments, is recommended. These instruments assess the biomechanical hazards present in work-related situations and monitor the effects of ergonomic improvements, without interfering with the environment^3^ ^,^ ^5^ ^,^ ^6^.

There are at least 30 observational instruments available in the literature, primarily in English, with varying purposes and approaches^5^. In Brazil, the Quick Exposure Check (QEC)^7^ ^,^ ^8^ has been the only observational instrument identified so far that examined the risk "*in loco*". The QEC was translated and adapted in addition to having its measurement properties tested.

The application of observational instruments can be conducted in one of two ways: through observation "*in loco*" or through filming^6^. The observation "*in loco*" depends on the observer's experience and is designed to evaluate static or repetitive postures. On the other hand, analysis with filming is reproducible and provides more details due to the possibility of analyzing the data repeatedly^6^ ^,^ ^9^. To determine the best approach, one must consider the following factors: the environment and individual that will be evaluated, costs, and the time available for evaluation^5^.

Among the tools available^5^, the Rapid Entire Body Assessment (REBA)^10^ ^,^ ^11^ is easy to understand, has a low implementation cost^9^ and has been used to evaluate different work environments and several biomechanical risk factors^12^ ^-^ ^16^. The REBA was created in 2000 by a team of physical therapists, ergonomists, occupational therapists and nurses through the encoding of 600 postural examples, with the purpose of assessing risky postures in the development of musculoskeletal injuries. The REBA differs from other instruments that aim to assess risky postures, such as the OWAS (Ovako Working Analysis System) and RULA (Rapid Upper Limb Assessment), by allowing analysis of a variety of tasks and by incorporating the weight of the object handled and the quality of the grip in the analysis^13^. Although the REBA was used to assess risk in several studies^12^ ^-^ ^14^, the original version of the instrument only had the percentage of inter-rater agreement tested, which ranged from 62% to 85%, excluding the category 'shoulder'^11^. However, the authors did not mention the statistical test used in the analysis.

Therefore, the aim of this study was to translate and cross-culturally adapt the REBA^10^ ^,^ ^11^ to Brazilian-Portuguese and analyze the intra- and inter-rater reliability.

## Method

To use the observational instrument in Brazil^5^, it is necessary to translate, cross-culturally adapt and test the measurement properties according to the set guidelines^15^ ^,^ ^16^ and address cultural and idiomatic differences.

The authors of the REBA^11^ instrument were contacted and authorized the translation and adaptation of the original instrument to Brazilian-Portuguese language.

The study was conducted in two stages: first, to translate and cross-cultural adapt the REBA^11^ instrument to the Brazilian-Portuguese, and second, to test the intra- and inter-rater reliability of the adapted version.

The present study was submitted to the Ethical Committee in Research from the Universidade da Cidade de São Paulo (UNICID), São Paulo, SP, Brazil and was approved under protocol N**º** 13668689. All participants signed the informed consent.

### Instrument

The REBA evaluated body segments and separated them into two groups (A and B). Group A consisted of the trunk, neck and legs, and has 60 postural combinations scored on Table A; group B consisted of the shoulder, forearm and wrist with 36 combinations to score on Table B.

The score of the load/force variable was added to the score found in Table A, resulting in the score A, and the score of the variable grip was added to the score found in Table B, resulting in the score B. The scores of A and B were crossed with each other in Table C, yielding the score C. The score from Table C was added to the score of the activity to generate the final REBA^11^ score. The final score was classified according to the level of risk that could range from negligible (1 point) to very high (11-15 points).

### Translation and cross-cultural adaptation of the REBA

The authors of the REBA instrument were consulted and authorized the translation and cross-cultural adaptation of the instrument. Translation and cross-cultural adaptation were conducted according to the guidelines proposed by Beaton et al.^16^, which included the following steps: translation, synthesis of the translation, back translation, expert committee and pre-test of the pre-final version.

The translation was performed by two independent Brazilians translators, both of whom were bilingual, who produced two versions in Brazilian-Portuguese (T1 and T2). Translator 1 had experience in occupational health and knowledge of the concepts of the instrument, while Translator 2 had no experience in health care and was not aware of the concepts of the instrument. This difference had the objective of improving conceptual and literary translation. The versions produced were synthesized by the expert committee into one single version (T12).

Next, the single version was translated back into the original language by two other independent, bilingual translators, who had no knowledge of the instrument. This step resulted in two back-translations (RT1 and RT2).

Throughout, the expert committee, which was composed of four physical therapists and two engineers working in the area of occupational health, evaluated all translations and back-translations (T1, T2, T12, RT1 and RT2). The committee verified the translations regarding the title, items, instructions, scores and record procedures and generated a pre-final version.

Ideally, according to the guidelines, the pre-test questionnaires should be applied to 30 or 40 patients or healthy individuals^16^. Therefore, we invited 90 health professionals via e-mail to participate in this process. However, only nine physical therapists, with general physical therapy background, agreed to evaluate the instrument. All of these evaluators assessed the risk of five tasks recorded on video. If the professionals reported difficulty in understanding any of the items of the instrument, the item was adjusted and reported to the expert committee^17^.

### Intra-and inter-rater reliability test

This test checks the intra- and inter-rater reliability of the REBA version adapted to Brazilian-Portuguese, following the guidelines proposed by Mokkink et al.^15^. Furthermore, we investigated the percentage of agreement among raters in the analysis.

The reliability test analyzed the behaviour of an instrument when used on a sample with repeated tests and represented the relative measurement error^18^ ^,^ ^19^. Reliability can be assessed by the intraclass correlation coefficient (ICC) or the Kappa coefficient (K), depending on the type of variable, and includes intra- and inter-rater reliability.

The intra-rater reliability measured the accuracy of the instrument when it was used more than once by the same evaluator. On the other hand, the inter-rater reliability measured the accuracy of the instrument used to evaluate a given task by different evaluators^19^. The score should be the same for the repeated measures.

In this study, the reliability was tested by two examiners who had five and seven years of experience in other research projects in the field of occupational health. They received instructions about the instrument and applied it in real situations by analyzing the video tasks. Over two consecutive days, eight hours of training was provided and directed toward understanding the application and the score of the instrument for analysing the tasks. After the training, the evaluators were placed in separate rooms to evaluate 53 video tasks of jobs from the textile industry, libraries, offices and supermarkets. The video tasks included: handling different objects and loading activities, prolonged static postures, repetition of movements, different positions and movement (e.g. squatting, walking, sweeping, typing, sewing).

The video tasks were recorded in real work situations from the third cycle of each task. The variation of the cycle time was analyzed using a digital stopwatch. The evaluators were told that they could replay the videos as many times as necessary to understand the tasks and analyse the movements. To facilitate this analysis, it was recommended that they identify the steps in each cycle and the percentage of time each posture was performed during the stages. The time used for each analysis was 8-10 minutes and the total time spent on the analysis of 53 video tasks to test and retest the evaluators was two weeks.

The REBA was evaluated and scored for each part of the body. This procedure was performed again after an interval of seven days. For inter-observer reliability analysis of only the first 53 video tasks were considered.

### Statistical analysis

The weighted kappa coefficient (Kw) was used for analysis of the categorical variables^20^. The linear weighted Kappa coefficient was classified, according to Landis and Koch^21^ as follows: almost perfect (>0.81), substantial (0.61 to 0.80), moderate (0.41 to 0.60), poor (0.21 to 0.40) and very poor (0.00 to 0.20).

The percentage of intra- and inter-rater reliability was assessed by cross tabulation (2×2) to calculate the percentage (%), with values from 0% to 100%. The higher the percentage, the better the agreement. All data were transferred to the software Statistical Package for Social Sciences (SPSS) version 17.0. The weighted Kappa test was performed in *VassarStats* statistical program.

## Results

### Translation and cross-cultural adaptation of the REBA

The versions derived from the translation stage (T1 and T2) and back translation (RT1 and RT2) stage were adequate, requiring only a few grammatical changes by replacing or substituting terms with synonyms for easy comprehension.

In the pre-test of the pre-final version, the nine physical therapy evaluators did not question any term related to the scores of groups A and B. The difficulties were in understanding the instructions for the completion of Tables A, B, and C and their association to the scores of load/force and grip. This could be observed in the following phrase that originally was: "For each region, there is a posture scoring scale notes plus adjustment for additional considerations. Then score the Load/Force and Coupling factors."

In the pre-final version used in the pre-test, the above sentence was as follows: "For every region, there is a score scale of posture plus the adjustment items for further considerations. Then, rate the Load/Force and Coupling factors." After the evaluators reported difficulty understanding the sentence, the necessary adjustments were made and the changes forwarded to the expert committee, resulting in the final adapted version of the REBA (Appendix 1S *). The phase was changed as follows: "For each region, there is a score scale of posture plus the adjustment items for additional considerations. Once you finish scoring group A, cross the scores in Table A, and once you finish scoring group B, cross the scores in Table B."

After the inclusion of the information in the adapted version, none of the evaluators had any difficulties with the instructions.

### Reliability assessment

The reliability tests were performed with the 95% confidence interval and showed that, for the intra-rater 1, the kappa index ranged from 0.129 for the item 'forearm' and 0.504 for the item 'trunk', representing very poor and moderate reliability, respectively. The percentage agreement was 15.09% for the item 'level of action' and 69.81% for the 'forearm' item (Table 1).

**Table 1:** Reliability test and percentage of agreement of the intra-rater 1 on the Brazilian version of the Rapid Entire Body Assessment (REBA).

Body region		
	Kappa linear weights (95% IC)	%Agreement
Trunk	0.504 (0.337 to 0.671)	54.71
Neck	0.326 (0.137 to 0.515)	52.83
Shoulder	0.236 (0.032 to 0.439)	45.28
Upper arm	0.129(0 to 0.381)	69.81
Wrist	0.316 (0.102 to 0.530)	54.71
Legs	0.292 (0.062 to 0.523)	52.83
Total	0.516 (0.291 to 0.688)	20.75
Level of Action	0.301 (0.119 to 0.483)	15.09

For the reliability of the intra-rater 2, the kappa index ranged from 0.104 for the item 'wrist' to 0.492 for the item 'legs', and -0.088 for the item 'forearm', representing very poor and moderate reliability, respectively. On the other hand, the correlations ranged from 18.86% for the item 'total score' to 62.26% for the 'forearms' and 'legs' items (Table 2).

**Table 2:** Reliability test and percentage of agreement of the intra-rater 2 on the Brazilian version of the Rapid Entire Body Assessment (REBA).

Body region		
	Kappa linear weights (95%IC)	%Agreement
Trunk	0.395 (0.241 to 0.548)	43.39
Neck	0.202 (0.016 to 0.388)	47.16
Shoulder	0.231 (0.070 to 0.393)	41.50
Upper arm	NC	62.26
Wrist	0.104 (0 to 0.310)	50.94
Legs	0.492 (0.292 to 0.693)	62.26
Total	NC	18.86
Level of action	NC	22.64

NC : The data could not be calculated by the statistical program.

For the reliability of the inter-raters 1 and 2, the kappa index ranged from 0.126 for the item 'neck' to 0.454 for the item 'legs', representing very poor and moderate reliability, respectively. The percentage of agreement ranged from 5.66% for the item 'level action' to 66.03% for the 'forearm' item (Table 3).

**Table 3:** Reliability test and percentage of agreement of the inter-rater 1 and 2 on the Brazilian version of the Rapid Entire Body Assessment (REBA).

Body region		
	Kappa linear weights (95%IC)	%Agreement
Trunk	0.307 (0.159 to 0.456)	32.07
Neck	0.126 (0 to 0.324)	35.84
Shoulder	NC	28.30
Upper arm	0.194 (0 to 0.472)	66.03
Wrist	NC	35.84
Legs	0.454 (0.285 to 0.624)	54.71
Total	NC	9.43
Level of Action	NC	5.66

NC : The data could not be calculated by the statistical program.

## Discussion

The evaluation of the work environment for risk factors that may affect the Brazilian workers is essential in the prevention and reduction of WMSD's. In Brazil, there are few tools available that are acceptable for this analysis, unlike what is available in other countries^5^.

Few observational instruments that have been translated and adapted to Brazilian-Portuguese can be used with Brazilian workers. Additionally, there are many studies that used observational instruments without translation and adaptation^12^ ^-^ ^14^. The use of a foreign instrument without its proper adaptation might jeopardize the validity and reliability of the evaluations conducted^22^.

The process of translation and adaptation is as important as the development of a new instrument^23^. In this process, one should always seek the highest possible equivalence between the original instrument and its translated and adapted version. In this context, we chose to translate, cross-culturally adapt to the Brazilian-Portuguese language and test the reliability of the REBA^10^ ^,^ ^11^ instrument.

The REBA is an instrument that evaluates the work environment for biomechanical risk factors and where workers might be exposed^10^ ^,^ ^11^. The process of translation and cross-cultural adaptation to Brazilian-Portuguese followed the model proposed by Beaton et al.^16^, and resulted in a version equivalent to the source instrument.

During the process of modifying the pre-test into the pre-final version of the REBA, difficulties were reported by the evaluators in the general instructions, which required the exchange, insertion and deletion of several words to make the tests clearer and easier to understand. However, the original version of the REBA^10^ ^,^ ^11^ itself is not very clear with regards to the instructions on the application, making it difficult to understand. The translated and adapted version of the REBA made the assessment of risks in the work environment factors more plausible after the inclusion of the additional information. However, we must recognize the limitation of the terms added since these additions were based only on nine evaluators' pre-test responses.

When analyzing the reliability test results of the translated and adapted instrument, we observed that the Brazilian-Portuguese version of the REBA presented reliability varying from very poor to moderate for intra- and inter-raters 1 and 2, with Kappa weighted values ranging from 0.104 for the item 'grip' to 0.504 for the item 'trunk'. The reliability property states that using an instrument repeatedly in a test with stable conditions should produce similar results^16^. Based on the results of this study, these conditions were not observed in this study.

The reliability values observed were below the values considered by the guideline that suggests reliability values be above 0.70^17^ ^,^ ^24^. Some aspects might have contributed to these low values, such as the characteristics of the REBA, insufficient training of the evaluators and the fact that the pre-test was performed by only nine physical therapists. A combination of factors could have contributed to these low reliability values. On the other hand, the mean percentage of agreement ranged from 5.66% for the item 'level action' to 69.81% for the item 'forearm'. The original version of the REBA only reported the values for percent agreement, ranging between 62-85%, for the inter-rater analysis^11^. These reliability values were higher than the values found in this study.

It should be noted that the REBA instrument has limitations, even in its native language, since there are no details of its reliability and only expresses values in percentage agreement. Reliability values below 0.70 have been observed in other instruments. For example, the Quick Exposure Check (QEC) instrument, translated and adapted to the Brazilian-Portuguese, showed values of moderate intra-rater reliability (0.41 to 0.60), and moderate to substantial inter-rater reliability (0.62 to 0.86)^7^. The fact that the reliability results of the QEC were slightly higher than the results of the REBA could be related to the differences in the scoring instruments. The QEC is more objective with less number of options for positions of each body segment.

Moreover, most observational instruments that assess risks and were described in a systematic review on the topic also presented reliability values below 0.70^5^.

When considering that these instruments represent one of the best ways available to assess occupational risk factors, it is necessary to interpret the results with caution. So far, only the reliability of the measurement properties was tested in the Brazilian-Portuguese version of the REBA due to the limitations found by the study, such as the difficulty of finding trained evaluators to observe at least 100 tasks - the adequate number of tasks to test, for example, for internal consistency. Another possible limitation of this study is the number of professionals who completed the pre-test of the pre-final version of the instrument, since according to the guidelines, it should have been conducted with a group of 30 to 40 individuals. Moreover, the improvement of the analysis is related to the training, thus implying that the low reliability results could indicate that the evaluators' training was insufficient, suggesting the need for further training compared to the one proposed. Finally, it is recommended that other studies re-assess the reliability and other properties, such as the validity and responsiveness.

## Conclusion

The REBA instrument showed satisfactory results in the translation and adaptation process. However, the instrument had a reliability ranging from poor to moderate and values below the guidelines recommended by the measurement of properties, indicating that reliability needs to be reassessed. Therefore, the use and data interpretation of the REBA should be conducted with caution, and future studies should aim to reassess the reliability and other measurement properties of the instrument.

## Footnotes

* Supplementary materials are available online at http://www.scielo.br/scielo.php?script=sci_issues&pid=1413-3555&lng=en&nrm=iso
